# Towards Bacteria Counting in DI Water of Several Microliters or Growing Suspension Using Impedance Biochips

**DOI:** 10.3390/bios10080082

**Published:** 2020-07-23

**Authors:** Mahdi Kiani, Astrid Tannert, Nan Du, Uwe Hübner, Ilona Skorupa, Danilo Bürger, Xianyue Zhao, Daniel Blaschke, Lars Rebohle, Charaf Cherkouk, Ute Neugebauer, Oliver G. Schmidt, Heidemarie Schmidt

**Affiliations:** 1 Department Nano Device Technology, Fraunhofer Institute for Electronic Nano Systems, Technologie-Campus 3, 09126 Chemnitz, Germany; danilo.buerger@enas.fraunhofer.de (D.B.); xianyue.zhao@enas.fraunhofer.de (X.Z.); 2 Material Systems for Nanoelectronics, Chemnitz University of Technology, 09126 Chemnitz, Germany; o.schmidt@ifw-dresden.de; 3 Leibniz Institute of Photonic Technology, Albert-Einstein-Str. 9, 07745 Jena, Germany; Astrid.Tannert@leibniz-ipht.de (A.T.); uwe.huebner@leibniz-ipht.de (U.H.); daniel.blaschke@leibniz-ipht.de (D.B.); ute.neugebauer@leibniz-ipht.de (U.N.); 4 Center for Sepsis Control and Care, Jena University Hospital, Am Klinikum 1, 07747 Jena, Germany; 5 Institute for Solid State Physics, Friedrich Schiller University Jena, 07743 Jena, Germany; 6 Helmholtz-Zentrum Dresden-Rossendorf, Bautzner Landstraße 400, 01328 Dresden, Germany; i.skorupa@hzdr.de (I.S.); l.rebohle@hzdr.de (L.R.); c.cherkouk@hzdr.de (C.C.); 7 Institute for Integrative Nanosciences IFW Dresden, Helmholtzstr. 20, 01069 Dresden, Germany

**Keywords:** bacterial cell counting, biochips, impedance spectroscopy, *Escherichia coli*, electrical equivalent circuit model

## Abstract

We counted bacterial cells of *E. coli* strain K12 in several-microliter DI water or in several-microliter PBS in the low optical density (OD) range (OD = 0.05–1.08) in contact with the surface of Si-based impedance biochips with ring electrodes by impedance measurements. The multiparameter fit of the impedance data allowed calibration of the impedance data with the concentration c_b_ of the *E. coli* cells in the range of c_b_ = 0.06 to 1.26 × 10^9^ cells/mL. The results showed that for *E. coli* in DI water and in PBS, the modelled impedance parameters depend linearly on the concentration of cells in the range of c_b_ = 0.06 to 1.26 × 10^9^ cells/mL, whereas the OD, which was independently measured with a spectrophotometer, was only linearly dependent on the concentration of the *E. coli* cells in the range of c_b_ = 0.06 to 0.50 × 10^9^ cells/mL.

## 1. Introduction

The techniques used to count biological species can be categorized into physical and biological ones. Physical methods will yield a total (living and dead) microorganisms count [1], while biological methods only record living organisms. A routinely used and easy physical way to count bacteria cells is the measurement of optical density (OD) at 600 nm [2]. Since this method measures the turbidity, and thus the scatter of light rather than the absorption, Beer’s law and the linear dependence of the OD on the cell number are only applicable in a certain narrow (usually highly diluted, i.e., 0.1 < OD < 1.0) range [3]. Thus, counting of biological species by OD measurements in a large concentration range produces some technical and interpretative problems because OD data saturate above a threshold concentration. Moreover, since the intensity reduction of the incident light due to scatter is highly dependent on the geometry of the instrumentation, OD values taken from the same suspension can differ by as much as a factor of 2, depending on the device used for measurement. For this reason, calibration of each device is required using suspensions with known cell numbers. Further parameters that might influence the reliability of OD measurements are changes in the scattering behavior of bacterial cells in different growth phases and under treatment with antibiotics, as well as ingredients of the growth medium, which change its refractive index [4]. To assess the exact number and species of bacteria in a suspension, the biological method of plate counting agar (PCA) [5] is used, which involves plating of serial dilutions of the relevant sample and counting the growing colonies on the next day. This method is very labor-intensive and time-consuming, giving results only after approximately 24 h. Alternative adequate, physical methods (e.g., microfluidic impedance cytometer [6] and impedance biochips [7,8]) have been used to count and discriminate biomaterial cells on the basis of their dielectric and electrical properties [9,10]. Impedance spectroscopy is one of the techniques used for the rapid counting of bacterial and other biological species [11]. It uses the conductance changes for quantitative and qualitative assessments of microbial growth [12]. Impedance spectroscopy was first proposed as an alternative method to replace the plate count technique for rapid screening of microbial content by Cady (1978) [13]. In our previous work, we counted different microorganisms (i.e., *Lysinibacillus sphaericus* JG-A12, in deionized (DI) water [14] and yeast *Saccharomyces cerevisiae* in deionized (DI) water and in glucose solution [15]) using impedance measurements on impedance biochips, where several microliters of liquid with the biological species was filled in the ring electrode area of the impedance biochip. We showed that the measured impedance for high OD is significantly different and systematically changes if up to 5 μL of the medium with or without microorganisms is added to 20 μL DI medium [14,15].

In the present work, we used the *Eschericha coli* (*E. coli*) strain K12 in the concentration range from 0.06 × 10^9^ to 1.26 × 10^9^ cells/mL and investigated the countability of *E. coli* in several-microliter liquid using impedance biochips. Virulent strains of Gram-negative *E. coli* are the main causative organisms of urinary tract infections and of urosepsis. In addition, *E. coli* can also cause certain gastroenterological diseases and neonatal meningitis. Additionally, certain non-pathologic strains of *E. coli*, including K12, are utilized in a variety of biotechnological and molecular biological applications and also serve as model organisms for a number of microbiological studies. For many of these applications, the number of cells in a growing suspension has to be determined. Detection of bacterial cell density is also an issue in the characterization of pathological patient isolates, for instance when determining antibiotic susceptibility.

We counted heat-inactivated immobilized *E. coli* cells using impedance biochips already filled with 20 µL of either DI water or PBS after adding 1, 2, 3, 4, and 5 μL of DI water or PBS, respectively; and after adding 1, 2, 3, 4, and 5 μL of *E. coli* suspended in DI water or PBS, respectively. We counted heat-inactivated *E. coli* cells using impedance biochips in DI water or PBS by subsequently adding a small amount of sample solution to the chips filled with 20 μL of solvent. The impedance change of the impedance biochips was measured, modeled, and correlated with the concentration of *E. coli* in the suspension.

## 2. Materials and Methods

### 2.1. Preparation of Bacteria and OD Measurements

**Figure 1:**
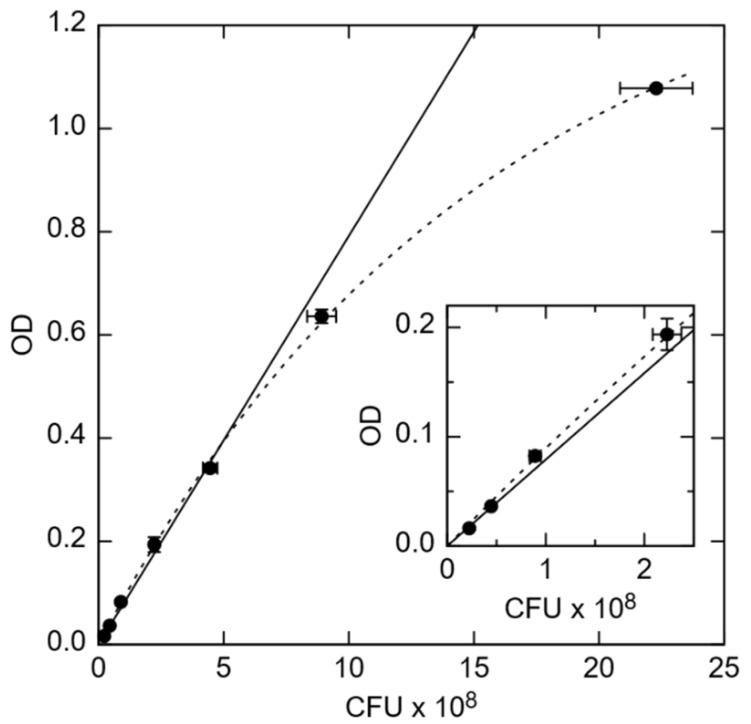
Optical density (OD) vs. colony-forming unit (CFU) counts of *E. coli* in units of 10^8^/mL. (Inset) Zoomed in data in the OD range from 0.00 to 0.22. The solid line is a linear extrapolation to larger cell concentrations, while the dashed line is an exponential fit to saturation at maximum. Error bars indicate the mean ± standard deviation of 2 PCA measurements (*x*-axis) and 3 OD measurements (*y*-axis).

*E. coli*, strain K12 have been used to investigate the countability of bacteria of small concentration in a several-microliter suspension. To assess the exact number of bacteria in the suspension (colony forming units, CFU) (*x*-axis in Figure 1) and to correlate the bacteria concentration with OD (*y*-axis in Figure 1), the PCA techniques were used for calibration. Therefore, *E. coli* K12 were cultivated overnight in tryptic soy broth (TSB, Carl Roth GmbH) at 37 °C while shaking with 160 revolutions per minute (rpm) under ambient air conditions. OD was determined at different dilutions of this overnight culture ranging from 1:2 to 1:200 in TSB in triplicate using the cuvette port of a microplate reader Spark^®^ 20M (Tecan, Männedorf, Switzerland).

The amount of viable CFUs in the overnight cultured solution was determined by the PCA technique. Therefore, a solution of OD = 0.1 was diluted to a concentration corresponding 1:10^5^ and 1:10^6^ in duplicate, respectively. Then, 100 μL of each dilution was plated onto an agar plate and cultivated at 37 °C overnight. Visible colonies were counted the next day. The OD depends linearly on the concentration of *E. coli* in the OD range from about 0.015 to 0.34, corresponding to 0.22 to 2.3 × 10^8^ CFU/mL, respectively. The optical density was determined at a 1:10 dilution in TSB in duplicate to determine the cell count. One microliter of the bacterial suspension was centrifuged at 13,000 xG for 90 s, the bacterial pellet was washed once in Dulbecco’s phosphate buffered saline (PBS, Merck Biochrome), and then resuspended in 1 mL PBS. Bacteria were heat-inactivated at 95 °C for at least 30 min. Heat-inactivated *E. coli* were washed twice in PBS diluted 1:2 in DI water (0.5× PBS) and resuspended in 0.5× PBS to about 12.5 × 10^8^ (low concentration) or 63 × 10^8^ cells/mL (moderate concentration).

### 2.2. Structural Description

**Figure 2:**
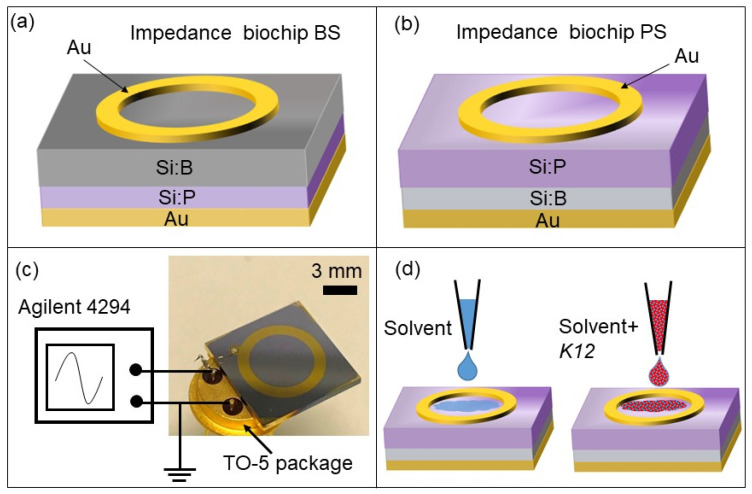
Schematic sketch of the (**a**) BS and (**b**) PS impedance biochips with a ring top electrode and with (**a**) boron ions (B) implanted into Si:P or with (**b**) phosphorus (P) ions implanted into Si:B. The top and bottom electrodes were wire-bonded to the pins of a TO-5 (Transistor Outline with base diameter of 8.9 mm) package (**c**) and connected to an Agilent 4294A impedance analyzer. A several-microliter solvent (i.e., DI water or PBS) and *E. coli* cells were added into the top ring electrode and the impedance of the biochip was measured.

To fabricate the BS (boron-implanted Samples) impedance biochips boron ions (B) were implanted into Si:P, and to fabricate the PS (Phosphorus-implanted Samples) impedance biochips phosphorus (P) ions were implanted into Si:B (Table 1). Figure 2a,b show the structures of the BS and PS impedance biochips, respectively. Approximately 150-nm-thick gold (Au) layers were deposited by DC magnetron sputtering on silicon wafers measuring 525 µm in thickness, while unstructured gold (Au) formed the bottom contact. Au ring electrodes with inner and outer diameters of 5.7 mm and 7.8 mm, respectively, were fabricated using a lift-off process.

A ring electrode was selected because of the homogenous field distribution between the ring top electrode and unstructured bottom electrode [16]. The detection limit for a small number of cells in a liquid by impedance measurements using the Si biochip strongly depends on the volume of liquid for which the top electrode structure is optimized.

The presented ring electrode structure was optimized with respect to the inner and outer ring diameters to sense suspension volumes between 10 and 30 μL. Dependent on the concentrations of the implanted dopants, the impedance biochips without liquid filling have a pn junction (boundary between a p-type and n-type semiconductor) capacitance and a capacitance for the Schotty contact formed by the Au ring electrode. The capacitances of the p + n junction in BS and of the n + p junction in PS amount to 4.8 × 10^−9^ F and 2.49 × 10^−9^ F, respectively. The capacitances of the Schottky contact formed by the Au ring electrodes on BS and PS amount to 3.12 × 10^−7^ F and 4.49 × 10^−8^ F, respectively. If a smaller suspension volume was detected, the inner and outer diameters of the ring electrodes would have to be reduced. The standard TO-5 package (Figure 2c) was used to assemble the impedance biochip and measurement setup. The top and bottom contacts of the biochips were bonded to the pins of this package and an Agilent 4294A precision impedance analyzer was utilized to record the impedance characteristics of the biochips by sweeping the frequency from 40 Hz to 1 MHz under normal daylight at room temperature. This impedance analyzer works in a range covering 10^−3^ to 10^8^ Ohm and is suitable for impedance spectroscopy of the investigated Si biochips with impedance changes observed in the range from 10^1^ Ohm up to 10^5^ Ohm. Before any measurement, the network analyzer was calibrated by performing 3 steps, namely shortcut, open circuit, and 50 Ohm calibration. Typically, one frequency sweep lasts 30 s, i.e., if the impedance data are calibrated with the cell concentration, impedance measurements for cell counting can be performed in less than 1 min. We chose two media liquids and added either DI water solvent and the *E. coli* or PBS solvent and *E. coli* into the gold ring top electrode region.

### 2.3. Modelling

**Figure 3:**
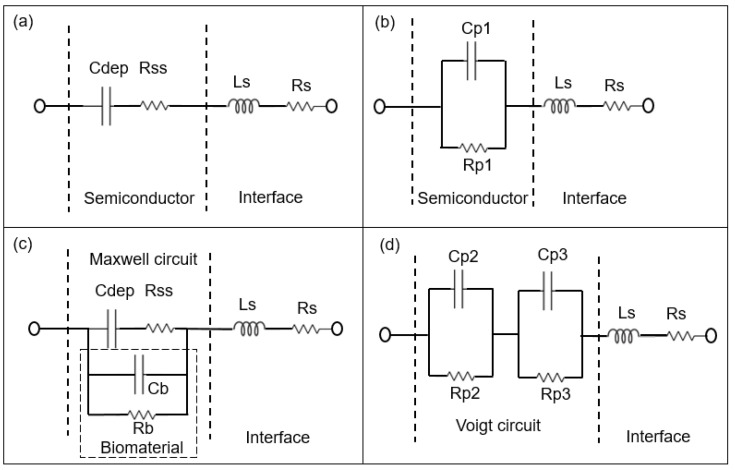
Schematic sketch of the equivalent circuit models of the BS biochip, where the capacitance of the Schottky contact formed by the Au ring electrode can be neglected without analyte (**a**) based on the physiochemical structure of the biochip and (**b**) based on the associated RC (Resistor and Capacitor) pairs. The parallel capacitor (Cp1) and parallel resistor (Rp1) can be transferred from Cdep (depletion capacitor) and Rss (semiconductor resistor) Cb and Rb corresponds to capacitive and resistive parts of biomaterial respectively. Cdep, Rss, Cb and Rb are transferred to the Parallel capacitor Cp2 and Cp3 and parallel resistor Rp2 na Rp3 from Equivalent circuit models of biochips with analyte in a (**c**) to Maxwell circuit and in a (**d**) Voigt circuit [14]. Ls and Rs are series inductor and resistor of the interface respectively.

**Figure 4:**
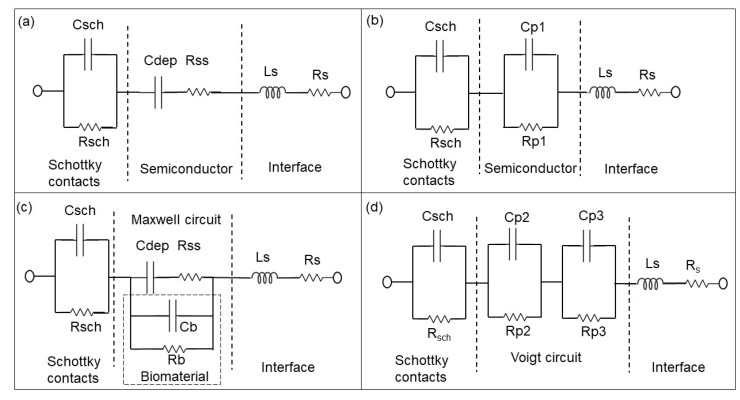
Equivalent circuit models of PS biochips (**a**) without solvent and bacteria and (**b**) based on the associated RC pairs. Equivalent circuit models of PS biochips with solvent or bacteria (**c**) and RC pairs (**d**) in Voigt fashion [14].

During impedance measurements, a small alternating current (AC) test signal of 50 mV was applied to BS and PS impedance biochips, while the complex resistance, i.e., the impedance, was measured for different test frequencies and plotted in the form of a Nyquist plot. This measurement technique is named impedance spectroscopy (ImS) [17]. The change of the recorded impedance data was correlated with the independently determined cell concentration (Figure 1). ImS helps to detect biological species in contact with the surface of the impedance biochip, because those biological species change the impedance of impedance biochips in the area inside the ring electrodes. The corresponding electrical equivalent circuit is obtainable based on the electrical properties from the recorded Nyquist plots of the biochips without and with *E. coli* K12 cells [18]. A Nyquist plot reveals the contribution of different components in the equivalent circuit [19]. As an example, an imperfect semicircle is correlated with constant phase elements (CPE) [20]. In the physical structure of the biochip, the capacitance and resistance are associated with space charge polarization regions and with adsorption of *E. coli* in the area inside the electrode. Usually, most of the structures with electrodes contain a geometrical capacitance and a bulk resistance in parallel to it [21,22,23]. For the used BS and PS biochips, the bulk capacitance of the p-n junction depletion region and the capacitance of the Schottky contacts between electrodes and semiconductor are modeled with resistor and capacitor pair [20]. To model the equivalent circuit parameters from the electrical equivalent circuit, complex non-linear least square (CNLS) software was used. Based on the Nyquist plot of the biochips. in which there are two imperfect semicircles, the electrical equivalent circuit shown in Figure 3b for the BS biochip and in Figure 4b for the PS biochip were selected [24,25].

The Rp2–Cp2 and Rp3–CP3 pairs in Figure 3d and Figure 4d correspond to the impedance of the pn junction below the Au ring electrode and below the contact formed by the liquid. Rsch and Csch describe the capacitance of the PS biochip due to the Schottky contact formed by the Au ring electrode (Figure 4c,d). The capacitance of the Schottky contact formed by the Au ring electrode (Figure 3c,d) on the BS biochip can be neglected. The Nyquist plot of the BS biochip without analyte consists of one semicircle. This semicircle can be represented by an associated resistor (R) and capacitor (C) pair, which is shown in Figure 3b. From the other side, the physical structures of the biochips consist of a p + n- junction at the boundary between p+ and n-type silicon. A depletion region is formed at the interface of these two semiconductor types, consisting of Cdep and Rss (Figure 3a,c). Thus, the BS biochip can be described by the introduced model in Figure 3a. This model can be transferred to electrical equivalent circuit shown in the Figure 3b by converting the series structures of Cdep and Rss to the parallel Cp1//Rp1 (Figure 3b), where Cp1 = Cdep·(Q^2^/(1 + Q^2^)), Rp1 = Rss·(1 + Q^2^) with Q = 1/(ω·Cdep·Rss) [26]

The Nyquist plot for the PS biochip without liquid, however, reveals two non-overlapping semicircles and a resistor–capacitor (RC) pair. These semicircles are due to the n + p- junction and the Schottky contacts that formed at the interfaces between Au top and bottom electrodes and the semiconductor. These metal–semiconductor Schottky contacts can be represented by an extra CPE and resistor pair in the electrical equivalent circuit [27] in Figure 4. The physiochemical model of the PS biochip, which is shown in Figure 4a, can then be transferred into Figure 4b by using series to parallel circuit exchange equations. After the DI water or PBS with *E. coli* is applied to the ring top electrode of the BS and PS biochips, an additional semicircle appears in the Nyquist plot. This semicircle is directly related to the cell concentration. In the electrical equivalent circuit with inserted liquid and with inserted liquid and *E. coli* in Figure 3d and Figure 4d, respectively, an additional R_3_C_3_ pair is used for modeling. This electrical modeling circuit needs to be equal to the physiochemical model. To transfer these two models, we employed two equivalent circuits that were equal at any frequencies. The Maxwell circuit in Figure 3c and Figure 4c can be transferred into the Voigt circuit, as illustrated in Figure 3d and Figure 4d.

In brief, the impedance change, which is dependent on the bacterial concentration, was modeled with 4 parameters, Rp2, Cp2, Rp3, and CP3. In a future work we will develop another model approach to fit the Cb and Rb of the liquid and of the liquid with the biomaterial directly in the Maxwell circuit. The parameters Rp2, Cp2, Rp3, and Cp3 depend on Cdep, Rss, Cb, and Rb. Therefore, a multiparameter is needed to quantify the concentrations of *E. coli* cells by using the BS and PS biochips (Table 1).

The electrical equivalent circuit model of the BS impedance biochip with no medium and no bacteria consists of a CPE in parallel with a resistor (Figure 3b), while the electrical equivalent circuit for BS biochips BS with medium and with *E. coli* cells consists of two pairs of CPEs and resistors (Figure 3b). The PS biochip without analyte is modeled using two pairs of CPEs in parallel with resistors (Figure 4b), while for the PS biochips with medium and with *E. coli* cells the circuit consists of three pairs of CPEs and resistors (Figure 4c). The equivalent circuit parameters Rs and Ls contribute to the lead impedances. Note that the equivalent circuit model shown in Figure 3b for the BS biochip and Figure 4c for the PS biochip were applied to model impedance changes of the BS and PS biochips after adding 1, 2, 3, 4, and 5 μL of medium with bacteria at low (0.06 to 0.25 × 10^9^ cells/mL) and moderate concentrations (0.29 to 1.26 × 10^9^ cells/mL).

## 3. Results and Discussion

**Figure 5:**
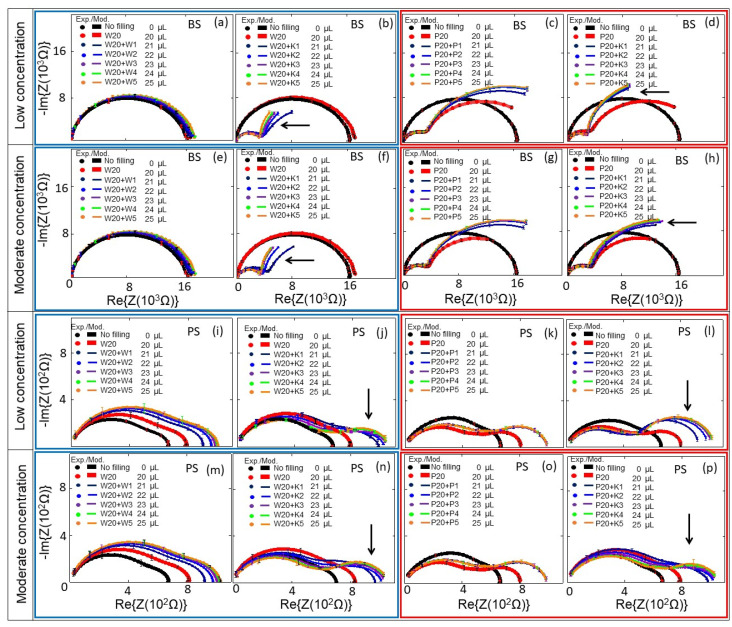
Experimental (symbols) and modeled (solid lines) Nyquist plots of the (**a**–**h**) BS and (**i**–**p**) PS biochips with no filling (black symbols and lines) and with 20 μL DI water/PBS (red symbols and lines), and with additional DI water/0.5× PBS with and without *E. coli* in steps of 1 μL: 1 μL (Navy), 2 μL (blue), 3 μL (violet), 4 μL (green), and 5 μL (orange). Here, 1 μL of *E. coli* and 5 μL of *E. coli* respectively correspond to bacterial cell concentrations of 0.06 × 10^9^/mL in 21 μL medium and 0.25 × 10^9^/mL in 25 μL medium for low concentrations; and of 0.29 × 10^9^/mL in 21 μL medium and to 1.26 × 10^9^/mL in 25 μL medium for moderate concentrations. The significant additional semicircles in the Nyquist plots, which are caused by the *E. coli*, are indicated by arrows. In the plots, blue boxes represent the results with DI water and red boxes represent the results 0.5× PBS. W represents DI water, P is short for 0.5× PBS, K stands for *E. coli* K12, and the numbers represent the volumes in microliters; the total volumes of the liquids on the top electrode are mentioned in the legend in microliters. The standard deviation error bars for 3 repeated measurements are also added to the measurement points.

*E. coli* cells were kept in DI water–0.5× PBS at cell concentrations of c_b_ = 0.06 to 0.25 × 10^9^ cells/mL (low concentration range, Figure 5b,d,j,l), and at cell concentrations of c_b_ = 0.29 to 1.26 × 10^9^ cells/mL (moderate concentration range, Figure 5f,h,n,p). The impedance changes of PS and BS biochips were studied under the same experimental conditions without any analyte in the area inside the top of the ring electrode, shown as black thick curves in Figure 5 (no filling). We used four BS impedance biochips and PS four impedance biochips (indicated by blue boxes for results with DI water and red boxes for 0.5× PBS in Figure 5). Impedance data in the same box were obtained by performing reference measurements without *E. coli* cells and measurements with *E. coli* cells on the same impedance biochip. First, we added 20 μL liquid without *E. coli* cells, plus 1, 2, 3, 4, and 5 μL liquid without *E. coli* cells on a given impedance biochip. After letting 25 μL of the liquid evaporate, the impedance of the impedance biochip without filling was remeasured and 20 μL liquid without *E. coli* was added and remeasured. Then, we added 1, 2, 3, 4, and 5 μL liquid with *E. coli* cells and measured the impedance. Note that the impedance of the biochips after evaporation of the liquid with *E. coli* cells is not the same as the impedance of the biochips after evaporation of the liquid without *E. coli* cells. The impedance values of biochips after adding 20 μL DI water without *E. coli* cells are shown in Figure 5a,b,e,f,i,j,m,n; and after adding 20 μL 0.5× PBS without *E. coli* cells are shown in Figure 5c,d,g,h,k,l,o,p. The impedance values of biochips after adding 1–5 μL DI water without *E. coli* cells are shown in Figure 5a,e,j,m; and after adding 1–5 μL 0.5× PBS without *E. coli* cells are shown in Figure 5c,g,k,o. Every filling step and corresponding impedance measurement lasted 1 min. Impedance values after adding 1–5 μL 0.5× PBS with *E. coli* cells to 20 μL DI water are shown in Figure 5b,f,j,n; and with 20 μL 0.5× PBS are shown in Figure 5d,h,l,p. Because of the different results for the impedance characteristics of the biochips with different media (e.g., Figure 5a,c), one can deduce that both biochips can be used to distinguish between these different media, namely DI water and 0.5× PBS, which is not possible with an optical microscope. Additionally, and more importantly, the impedance biochips can also be used to count adsorbed cells. Adsorbed cells cause a significant change in the impedance of the biochips. The impedance characteristics of the biochips with DI water or 0.5× PBS and the corresponding impedance characteristics of the biochips with *E. coli* (Figure 5) confirm the significant sensitivity of the biochips to adsorbed *E. coli* cells in the area inside the ring electrode. Remarkably, an additional significant semicircle forms in the impedance plot of the biochips with added *E. coli*. This additional semicircle is pointed out by an arrow in Figure 5b, d, f, h, j, l, n, p. We modeled the experimental impedance data and analyzed the modeled equivalent circuit parameters dependent on the filling of the area inside the ring electrode. The equivalent circuits for the biochips with DI water/0.5× PBS and for the BS biochip with dispensed *E. coli* in DI water/0.5× PBS are shown in Figure 3a,b, respectively.

Without solvent and bacteria, the equivalent circuit consists of two imperfect capacitors or CPEs (Cp1, Cp2), two parallel resistors (Rp1, Rp2), a contact resistor (Rs), and a contact inductor (Ls). The modeled impedance circuit parameters are listed in Appendix A Table A1, Table A2, Table A3, Table A4, Table A5, Table A6, Table A7, Table A8, Table A9, Table A10, Table A11 and Table A12. The equivalent circuits of the impedance spectra of the biochips with and without E. coli cells are different due to the additional appearance of the semicircles, which are indicated by arrows in Figure 5.

Accordingly, based on the experimental impedance characteristics of the biochips after adding analyte, the composition and cell numbers of the *E. coli* added to the biochips can be determined by modeling the parameters of the equivalent circuit shown in Figure 3b for the BS biochip, which consists of two imperfect capacitors (Cp1, Cp2); and those shown in Figure 4b for the PS biochip, which consists of three imperfect capacitors (Cp1, Cp2, Cp3) and three resistors (Rp1, Rp2, Rp3), a contact resistor (Rs), and a contact inductor (Ls).

**Figure 6:**
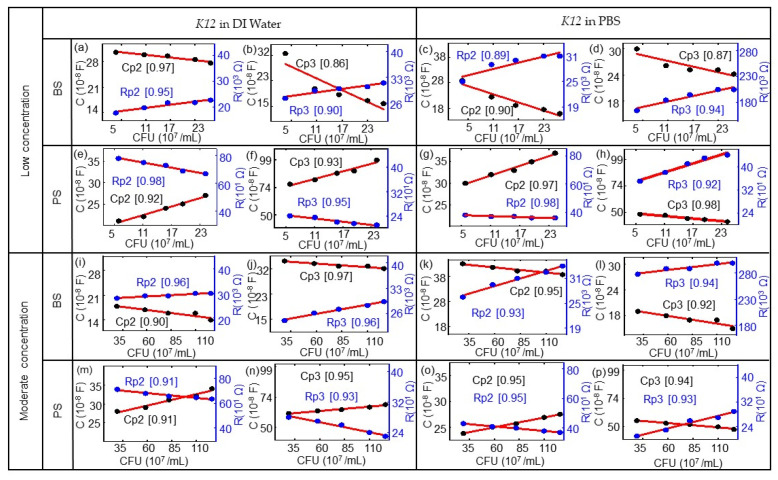
Modeled equivalent circuit parameters (dots) and linear fitting curves (red lines) for (**a**,**c**,**e**,**g**) Rp1 and Cp1 and for (**b**,**d**,**f**,**h**) Rp3 and CP3 for the BS biochip, dependent on the number of cells in 10^7^/mL in DI water or PBS. Modeled equivalent circuit parameters (dots) and linear fitting curves (red lines) for (**i**,**k**,**m**,**o**) Rp1 and Cp1 and for (**j**,**l**,**n**,**p**) Rp3 and CP3 for the PS biochip, dependent on the cell number in units of 10^7^/mL with the DI water and PBS media. The quality of the linear fitting was measured by the R-square or coefficient of determination (COD), as shown in the brackets for each data set. (This value is between 0 and 1; if the value is close to 1, the relationship between a data point and a line will be regarded as very strong.).

The corresponding ImS modeling results of the biochips based on Table A1, Table A2, Table A3, Table A4, Table A5, Table A6, Table A7 and Table A8 are shown in Figure 6.The *E. coli* cell concentration was determined by measuring the OD of a diluted stock solution and calculating the corresponding number of cells using the calibration data from Figure 1. Observed impedance changes can be related to concentrations of *E. coli* cells for low concentrations (0.06 × 10^9^ to 0.25 × 10^9^/mL) and moderate concentrations (0.29 to 1.26 × 10^9^/mL). The modeled equivalent circuit elements Rp2, Rp3, Cp2, and Cp3 depend linearly on the concentration of *E. coli* cells. There is a linear relationship with the nominal number of bacterial cells extracted for the biochips with low concentrations (0.06 × 10^9^/mL to 0.25 × 10^9^/mL) and with moderate concentrations (0.29 × 10^9^/mL to1.26 × 10^9^/mL). The modeling parameters Rp1 and Cp1 represent the Schottky contact at the electrode–semiconductor interface. If the size of the contact area is denoted as A, by adding the *E. coli* suspension to the top electrode region of the biochips, the effective area of the top contact is increased based on the equation Rp2 = ρ(d/A), where d denotes the thickness of the Schottky barrier and the resistance is reversely related to area A. Thus, there is a reduction in resistance Rp2 by adding the bacterial suspension. If we consider Cp2 = ε(A/d), with ε as the permittivity of the semiconductor, the relationship between Cp2 and A results in an increasing Cp2 with the increasing number of *E. coli* in the suspension.

## 4. Conclusions

We counted *E. coli* cells with concentrations from c_b_ = 0.06 × 10^9^/mL to c_b_ = 1.26 × 10^9^/mL in DI water and in PBS by analyzing the impedance of two different Si-based impedance biochips whose surfaces were in contact with the *E. coli* cells. Such impedance biochips are useful for disturbance-free monitoring of the cultivation of *E. coli* cells, providing significant time efficiency and high accuracy for a promising large range of cell concentrations. The significant changes between the impedance spectra before and after addition of the *E. coli* suspension into the top electrode region of the impedance biochips were evaluated. Developing a two-phase electrode structure helped to determine a proper equivalent circuit for the biochips with added bacteria. The modeling parameters Rp2, Cp2, Rp3, and Cp3 show the linear relationship with the *E. coli* cell number. Such linearly-dependent parameters were used to quantify the *E. coli* concentration. In comparison with impedance data, the dynamic range of OD data with respect to the cell concentration is smaller. OD data are only linear in the range of 0.06–0.5 × 10^9^ cells/mL. In the future, detection of the dead and living biomaterials will be studied for different cell sizes and concentrations, and the further applications in microbiology, food industry, and medicine will be evaluated.

## Figures and Tables

**Table 1:** Implantation parameters of phosphorus-implanted (phosphorus into Si:B) PS biochip and of boron-implanted (boron into Si:P) BS biochip. The Au ring top electrodes and unstructured Au bottom contacts were prepared after ion implantation.

Biochip	Implanted Ion	Ion Energy (MeV)	Ion Fluence (cm^−2^)
**PS**	Phosphorus	1.00	3 × 10^13^
**BS**	Boron	0.45	3 × 10^13^

**Figure A1:**
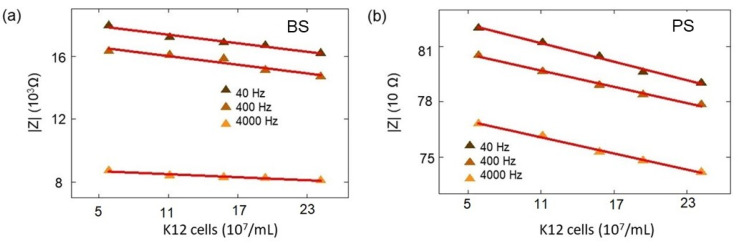
Impedance measured at test frequencies of 40 Hz, 400 Hz, and 4000 Hz on (**a**) BS (Table A13) and (**b**) PS (Table A14) biochips, dependent on the number of low-concentration *K12* bacteria and under normal illumination.

## Appendix A

**Table A1:** Modeled impedance parameters Cp2, Rp2, Cp3, Rp3, Rs, and Ls of the BS biochip with DI water (Figure 3 d). W20 + W0 = 20 µL DI water, W20 + W1 = 21 µL DI water, W20 + W2 = 2 µL DI water, W20+ W3 = 23 µL DI water, W20 + W4 = 24 µL DI water, W20 + W5 = 25 µL DI water, and Rs = 1.6 × 10^−9^ Ω and Ls = 2.6 × 10^−15^ H. BS biochip without DI water (Figure 3b) Cp1= 4.8 × 10^−9^ F, Rp1 = 1.6 ×10^4^ Ω, Rs = 1.6 × 10^- 9^ Ω, and Ls = 2.6 × 10^−15^ H. The calculated capacitance of the p-n junction in BS amounts to 3.7 × 10^−9^ F, which is comparable to CP1.

Circuit Element	Cp2 (F)	Rp2 (Ω)	Cp3 (F)	Rp3 (Ω)
BS + W20 + W0	6.2 × 10^−9^	1.7 × 10^4^	4.1 × 10^−7^	1.31× 10^4^
BS + W20 + W1	6.0 × 10^−9^	1.9 × 10^4^	4.3 × 10^−7^	1.3 × 10^4^
BS + W20 + W2	5.9 × 10^−9^	2.0 × 10^4^	4.9 × 10^−7^	1.4 × 10^4^
BS + W20 + W3	5.8 × 10^−9^	2.1 × 10^4^	5.5 × 10^−7^	1.6 × 10^4^
BS + W20 + W4	5.7 × 10^−9^	2.2 × 10^4^	5.8 × 10^−7^	1.8 × 10^4^
BS + W20 + W5	5.6 × 10^−9^	2.3 × 10^4^	6.0 × 10^−7^	1.9 × 10^4^

**Table A2:** Modeled impedance parameters Cp2, Rp2, Cp3, Rp3, Rs, and Ls of the BS biochip with DI water and low-concentration *K12* (Figure 5b). Here, c_b_ = 0.06 × 10^9^ to 0.25 × 10^9^ cells/mL, Cp1= 4.8 × 10^−9^ F, Rp1= 1.6 ×10^4^ Ω, Rs =1.6 × 10^−9^ Ω, and Ls = 2.6 × 10^−15^ H.

Circuit Element	Cp2 (F)	Rp2 (Ω)	Cp3 (F)	Rp3 (Ω)
BS + W20 + W0	6.2 × 10^−9^	1.7 × 10^4^	4.1 × 10^−7^	1.31× 10^4^
BS + W20 + K1	3.1 × 10^−9^	1.8 × 10^4^	3.3 × 10^−7^	2.4 × 10^4^
BS + W20 + K2	3.0 × 10^−9^	2.0 × 10^4^	2.1 × 10^−7^	3.0 × 10^4^
BS + W20 + K3	3.0 × 10^−9^	2.2 × 10^4^	1.9 × 10^−7^	3.0 × 10^4^
BS + W20 + K4	2.9 × 10^−9^	2.2 × 10^4^	1.7 × 10^−7^	3.1 × 10^4^
BS + W20 + K5	2.8 × 10^−9^	2.3 × 10^4^	1.6 × 10^−7^	3.2 × 10^4^

W20 = 20 μL DI water, K1 = 1 μL *K12* (c_b_ = 0.06 × 10^9^ cells/mL), K2 = 2 μL *K12* (c_b_ = 0.11 × 10^9^ cells/mL), K3 = 3 μL*K12* (c_b_ = 0.16 × 10^9^ cells/mL), K4 = 4 μL *K12* (c_b_ = 0.21 × 10^9^ cells/mL), K5 = 5 μL *K12* (c_b_ = 0.25 × 10^9^ cells/mL).

**Table A3:** Modeled impedance parameters Cp2, Rp2, Cp3, Rp3, Rs, and Ls of the BS biochip, with DI water and moderate-concentration *K12* (Figure 5f). here, 0.29 × 10^9^ to 1.26 × 10^9^ cells/mL, Rs = 1.6 × 10^−9^ Ω, and Ls =2.6 × 10^−15^ H.

Circuit Element	Cp2 (F)	Rp2 (Ω)	Cp3 (F)	Rp3 (Ω)
BS + W20 + W0	6.2 × 10^−9^	1.7 × 10^4^	4.1 × 10^−7^	1.31× 10^4^
BS + W20 + K1	3.0 × 10^−9^	3.0 × 10^4^	2.1 × 10^−7^	3.4 × 10^4^
BS + W20 + K2	2.9 × 10^−9^	3.2 × 10^4^	2.0 × 10^−7^	3.7 × 10^4^
BS + W20 + K3	2.8 × 10^−9^	3.5 × 10^4^	1.8 × 10^−7^	3.8 × 10^4^
BS + W20 + K4	2.6 × 10^−9^	4.2 × 10^4^	1.7 × 10^−7^	3.9 × 10^4^
BS + W20 + K5	2.5 × 10^−9^	4.7 × 10^4^	1.6 × 10^−7^	4.0 × 10^4^

W20 = 20 μL DI water, K1 = 1 μL *K12* (c_b_ = 0.23 × 10^9^ cells/mL), K2 = 2 μL *K12* (c_b_ = 0.45 × 10^9^ cells/mL), K3 = 3 μL*K12* (c_b_ = 0.65 × 10^9^ cells/mL), K4 = 4 μL *K12* (c_b_ = 0.88 × 10^9^ cells/mL), K5 = 5 μL *K12* (c_b_ = 1.26 × 10^9^ cells/mL).

**Table A4:** Modeled impedance parameters Cp2, Rp2, Cp3, Rp3, Rs, and Ls of the BS biochip with PBS14 (Figure 5c), Rs =1.6 × 10^−9^ Ω, and Ls = 2.6 × 10^−15^ H.

Circuit Element	Cp2 (F)	Rp2 (Ω)	Cp3 (F	Rp3 (Ω)
BS + P20 + P0	2.0 × 10^−9^	3.3 × 10^4^	1.9 × 10^−7^	2.4× 10^4^
BS + P20 + P1	2.0 × 10^−9^	3.3 × 10^4^	2.2 × 10^−7^	2.6 × 10^4^
BS + P20 + P2	2.1 × 10^−9^	3.4 × 10^4^	2.3 × 10^−7^	2.7 × 10^4^
BS + P20 + P3	2.2 × 10^−9^	3.4 × 10^4^	2.4 × 10^−7^	2.8 × 10^4^
BS + P20 + P4	2.3 × 10^−9^	3.6 × 10^4^	2.5 × 10^−7^	2.9 × 10^4^
BS + P20 + P5	2.5 × 10^−9^	3.7 × 10^4^	2.7 × 10^−7^	3.0 × 10^4^

P20 = 20 μL PBS14 varying analyte: P1 = 1 μL PBS, P2 = 2 μL PBS, P3 = 3 μL PBS, P4 = 4 μL PBS, P5 = 5 μL PBS.

**Table A5:** Modeled impedance parameters Cp2, Rp2, Cp3, Rp3, Rs, and Ls of the BS biochip with PBS14 as the medium and low-concentration *K12* (Figure 5d), c_b_ = 0.06 × 10^9^ to 0.25 × 10^9^ cells/mL, Rs = 1.6 × 10^−9^ Ω, and Ls =2.6 × 10^−15^ H.

Circuit Element	Cp2 (F)	Rp2 (Ω)	Cp3 (F)	Rp3 (Ω)
BS + P20 + P0	2.0 × 10^−9^	3.3 × 10^4^	1.9 × 10^−7^	2.4× 10^4^
BS + P20 + K1	2.5 × 10^−9^	2.4 × 10^4^	3.1 × 10^−7^	1.8 × 10^4^
BS + P20 + K2	2.1 × 10^−9^	2.8 × 10^4^	2.7 × 10^−7^	2.0 × 10^4^
BS + P20 + K3	1.9 × 10^−9^	2.9 × 10^4^	2.6 × 10^−7^	2.1 × 10^4^
BS + P20 + K4	1.8 × 10^−9^	3.0 × 10^4^	2.6 × 10^−7^	2.2 × 10^4^
BS + P20 + K5	1.7 × 10^−9^	3.0 × 10^4^	2.5 × 10^−7^	2.2 × 10^4^

P20 = 20 μL PBS, K1 = 1 μL K12 (cb = 0.06 × 109 cells/mL), K2 = 2 μL K12 (cb = 0.11 × 109 cells/mL), K3 = 3 μLK12 (cb = 0.16 × 109 cells/mL), K4 = 4 μL K12(cb = 0.21 × 109 cells/mL), K5 = 5 μL K12 (cb = 0.25 × 109 cells/mL).

**Table A6:** Modeled impedance parameters Cp2, Rp2, Cp3, Rp3, Rs, and Ls of the BS biochip with PBS14 as the medium and moderate-concentration *K12* (Figure 5h), c_b_ =0.29 × 10^9^ to 1.26 × 10^9^ cells/mL, Rs = 1.6 × 10^−9^ Ω, and Ls =2.6 × 10^−15^ H.

Circuit Element	Cp2 (F)	Rp2 (Ω)	Cp3 (F)	Rp3 (Ω)
BS + P20 + P0	2.0 × 10^−9^	3.3 × 10^4^	1.9 × 10^−7^	2.4× 10^4^
BS + P20 +K1	1.9 × 10^−9^	2.8 × 10^4^	3.8 × 10^−7^	2.5 × 10^4^
BS + P20 +K2	1.8 × 10^−9^	2.9 × 10^4^	3.7 × 10^−7^	2.7 × 10^4^
BS + P20 +K3	1.7 × 10^−9^	2.9 × 10^4^	3.6 × 10^−7^	2.8 × 10^4^
BS + P20 +K4	1.7 × 10^−9^	3.0 × 10^4^	3.6 × 10^−7^	2.9 × 10^4^
BS + P20 +K5	1.5 × 10^−9^	3.0 × 10^4^	3.5 × 10^−7^	3.0 × 10^4^

P20 = 20 μL PBS, K1 = 1 μL K12 (cb = 0.23 × 109 cells/mL), K2 = 2 μL K12 (cb = 0.45 × 109 cells/mL), K3 = 3 μLK12 (cb = 0.65 × 109 cells/mL), K4 = 4 μL K12 (cb = 0.88 × 109 cells/mL), K5 = 5 μL K12 (cb = 1.26 × 109 cells/mL).

**Table A7:** Modeled impedance parameters Cp2, Rp2, Csch, Rsch, Cp3, Rp3, Rs, and Ls of the PS biochip with added DI water: W20 + W0 = 20 µL DI water, W20 + W5 = 25 μL DI water (Figure 5i), Rs = 2.5 × 10^−8^ Ω, and Ls = 2.6 × 10^−15^ H. PS without DI water (Figure 4d). Csch = 1.19 × 10^−7^ F and Rsch = 5.5 × 10^2^ Ω, Cp1= 2.49× 10^−9^ F and Rp1= 5.5 × 10^2^ Ω. The calculated capacitance of the p-n junction in PS amounts to 6.8 × 10^−9^ F and is comparable to CP1. The calculated capacitance of the Schottky contact in PS amounts to 4.49 × 10^−8^ F and is comparable to Csch. Rs = 2.5 × 10^−8^ Ω and Ls = 2.6 × 10^−15^ H.

Circuit Element	Cp2 (F)	Rp2 (Ω)	Csch (F)	Rsch (Ω)	Cp3 (F)	Rp3 (Ω)
PS + W20 + W0	5.9 × 10^−9^	7.4 × 10^2^	1.7 × 10^−7^	5.5 × 10^2^	2.2 × 10^−9^	2.4 × 10^2^
PS + W20 + W1	5.7 × 10^−9^	8.2 × 10^2^	1.4 × 10^−7^	5.5 × 10^2^	1.9 × 10^−9^	2.8 × 10^2^
PS + W20 + W2	5.5 × 10^−9^	8.5 × 10^2^	1.4 × 10^−7^	5.5 × 10^2^	1.7 × 10^−9^	3.1 × 10^2^
PS + W20 + W3	5.4 × 10^−9^	8.7 × 10^2^	1.3 × 10^−7^	5.4 × 10^2^	1.6 × 10^−9^	3.5 × 10^2^
PS + W20 + W4	5.2 × 10^−9^	8.9 × 10^2^	1.3 × 10^−7^	5.4 × 10^2^	1.5 × 10^−9^	3.8 × 10^2^
PS + W20 + W5	5.0 × 10^−9^	9.1 × 10^2^	1.3 × 10^−7^	5.4 × 10^2^	1.2 × 10^−9^	4.1 × 10^2^

W20 = 20 μL DI water, W1 = 1 μL DI water, W2 = 2 μL DI water, W3 = 3 μL DI water, W4 = 4 μL DI water, W5 = 5 μL DI water.

**Table A8:** Modeled impedance parameters Cp2, Rp2, Csch, Rsch, Cp3, Rp3, Rs, and Ls of the PS biochip with DI water and low-concentration *K12* (Figure 5j). Here, c_b_ = 0.06 × 10^9^ to 0.25 × 10^9^ cells/mL. Rs = 2.5 × 10^−8^ Ω and Ls = 2.6 × 10^−15^ H.

Circuit Element	Cp2 (F)	Rp2 (Ω)	Csch (F)	Rsch (Ω)	Cp3 (F)	Rp3 (Ω)
PS + W20 + K0	5.9 × 10^−9^	7.4 × 10^2^	1.7 × 10^−7^	5.5 × 10^2^	2.2 × 10^−9^	2.4 × 10^2^
PS + W20 + K1	2.1 × 10^−9^	7.8 × 10^2^	1.4 × 10^−7^	5.5 × 10^2^	7.8 × 10^−9^	2.4 × 10^2^
PS + W20 + K2	2.2 × 10^−9^	7.5 × 10^2^	1.4 × 10^−7^	5.5 × 10^2^	8.2 × 10^−9^	2.4 × 10^2^
PS + W20 + K3	2.4 × 10^−9^	7.3 × 10^2^	1.3 × 10^−7^	5.4 × 10^2^	8.8 × 10^−9^	2.2 × 10^2^
PS + W20 + K4	2.5 × 10^−9^	6.9 × 10^2^	1.3 × 10^−7^	5.4 × 10^2^	9.0 × 10^−9^	2.1 × 10^2^
PS + W20 + K5	2.7 × 10^−9^	6.7 × 10^2^	1.3 × 10^−7^	5.4 × 10^2^	10 × 10^−9^	2.1 × 10^2^

W20 = 20 μL DI water, K1 = 1 μL K12 (cb = 0.06 × 109 cells/mL), K2 = 2 μL K12 (cb = 0.11 × 109 cells/mL), K3 = 3 μL K12 (cb = 0.16 × 109 cells/mL), K4 = 4 μL K12 (cb = 0.21 × 109 cells/mL), K5 = 5 μL K12 (cb = 0.25 × 109 cells/mL).

**Table A9:** Modeled impedance parameters Cp2, Rp2, Csch, Rsch, Cp3, Rp3, Rs, and Ls of the PS biochip with DI water and moderate-concentration *K12* (Figure 5n), where c_b_ = 0.29 × 10^9^ to 1.26 × 10^9^ cells/mL. Rs = 2.5 × 10^−8^ Ω and Ls = 2.6 × 10^−15^ H.

Circuit Element	Cp2 (F)	Rp2 (Ω)	Cpsch (F)	Rsch (Ω)	Cp3 (F)	Rp3 (Ω)
PS + W20 + K0	5.9 × 10^−9^	7.4 × 10^2^	1.7 × 10^−7^	5.5 × 10^2^	2.2 × 10^−9^	2.4 × 10^2^
PS + W20 + K1	2.8 × 10^−9^	6.8 × 10^2^	1.4 × 10^−7^	5.5 × 10^2^	6.1 × 10^−9^	2.8 × 10^2^
PS + W20 + K2	2.9 × 10^−9^	6.5 × 10^2^	1.4 × 10^−7^	5.5 × 10^2^	6.3 × 10^−9^	2.7 × 10^2^
PS + W20 + K3	3.1 × 10^−9^	6.3 × 10^2^	1.3 × 10^−7^	5.4 × 10^2^	6.4 × 10^−9^	2.6 × 10^2^
PS + W20 + K4	3.2× 10^−9^	6.2 × 10^2^	1.3 × 10^−7^	5.4 × 10^2^	6.6 × 10^−9^	2.4 × 10^2^
PS + W20 + K5	3.4 × 10^−9^	6.1 × 10^2^	1.3 × 10^−7^	5.4 × 10^2^	6.8 × 10^−9^	2.3 × 10^2^

W20 = 20 μL DI water, K1 = 1 μL K12 (cb = 0.23 × 109 cells/mL), K2 = 2 μL K12 (cb = 0.45 × 109 cells/mL), K3 = 3 μLK12 (cb = 0.65 × 109 cells/mL), K4 = 4 μL K12 (cb = 0.88 × 109 cells/mL), K5 = 5 μL K12 (cb = 1.26 × 109 cells/mL).

**Table A10:** Modeled impedance parameters Cp2, Rp2, Csch, Rsch, Cp3, Rp3, Rs, and Ls of the PS biochip with PBS (Figure 5k). Rs = 2.5 × 10^−8^ Ω and Ls = 2.6 × 10^−15^ H.

Circuit Element	Cp2 (F)	Rp2 (Ω)	Cpsch (F)	Rsch (Ω)	Cp3 (F)	Rp3 (Ω)
PS + P20 + P0	7.8 × 10^−9^	4.7 × 10^2^	2.7 × 10^−8^	3.5 × 10^2^	1.4 × 10^−7^	2.8 × 10^2^
PS + P20 + P1	7.9 × 10^−9^	6.8 × 10^2^	1.4 × 10^−8^	5.5 × 10^2^	1.1 × 10^−9^	3.4 × 10^2^
PS + P20 + P2	7.9 × 10^−9^	6.7 × 10^2^	1.4 × 10^−8^	5.4 × 10^2^	1.2 × 10^−9^	3.4 × 10^2^
PS + P20 + P3	7.9 × 10^−9^	6.7 × 10^2^	1.4 × 10^−8^	5.4 × 10^2^	1.2 × 10^−9^	3.4 × 10^2^
PS + P20 + P4	7.9 × 10^−9^	6.7 × 10^2^	1.4 × 10^−8^	5.4 × 10^2^	1.2 × 10^−9^	3.4 × 10^2^
PS + P20 + P5	7.9 × 10^−9^	6.7 × 10^2^	1.4 × 10^−8^	5.4 × 10^2^	1.2 × 10^−9^	3.4 × 10^2^

P20 = 20 μL PBS with varying analyte levels: P1 = 1 μL PBS, P2 = 2 μL PBS, P3 = 3 μL PBS, P4 = 4 μL PBS, P5 = 5 μL PBS.

**Table A11:** Modeled impedance parameters Cp2, Rp2, Csch, Rsch, Cp3, Rp3, Rs, and Ls of the PS biochip with PBS14 and low-concentration *K12* (Figure 5l), c_b_ =0.06 × 10^9^ to 0.25 × 10^9^ cells/mL. Rs = 2.5 × 10^−8^ Ω and Ls =2.6 × 10^−15^ H.

Circuit Element	Cp2 (F)	Rp2 (Ω)	Cpsch (F)	Rsch (Ω)	Cp3 (F)	Rp3 (Ω)
PS + P20 + K0	7.8 × 10^−9^	4.7 × 10^2^	2.7 × 10^−8^	3.5 × 10^2^	1.4 × 10^−7^	2.8 × 10^2^
PS + P20 + K1	3.0 × 10^−9^	3.8 × 10^2^	1.4 × 10^−7^	5.5 × 10^2^	4.8 × 10^−9^	3.5 × 10^2^
PS + P20 + K2	3.2 × 10^−9^	3.7 × 10^2^	1.3 × 10^−7^	5.4 × 10^2^	4.7 × 10^−9^	3.8 × 10^2^
PS + P20 + K3	3.3 × 10^−9^	3.7 × 10^2^	1.3 × 10^−7^	5.4 × 10^2^	4.4 × 10^−9^	4.1 × 10^2^
PS + P20 + K4	3.5 × 10^−9^	3.6 × 10^2^	1.3 × 10^−7^	5.4 × 10^2^	4.3 × 10^−9^	4.3 × 10^2^
PS + P20 + K5	3.7 × 10^−9^	3.6 × 10^2^	1.4 × 10^−7^	5.5 × 10^2^	4.1 × 10^−9^	4.4 × 10^2^

P20 = 20 μL PBS, K1 = 1 μL K12 (cb = 0.06 × 109 cells/mL), K2 = 2 μL K12 (cb = 0.11 × 109 cells/mL), K3 = 3 μL K12 (cb = 0.16 × 109 cells/mL), K4 = 4 μL K12(cb = 0.21 × 109 cells/mL), K5 = 5 μL K12 (cb = 0.25 × 109 cells/mL).

**Table A12:** Modeled impedance parameters Cp2, Rp2, Csch, Rsch, Cp3, Rp3, Rs, and Ls of the PS biochip with PBS14 and moderate-concentration *K12* (Figure 5p), c_b_ =0.29 × 10^9^ to 1.26 × 10^9^ cells/mL. Rs = 2.5 × 10^−8^ Ω and Ls =2.6 × 10^−15^ H.

Circuit Element	Cp2 (F)	Rp2 (Ω)	Cpsch (F)	Rsch (Ω)	Cp3 (F)	Rp3 (Ω)
PS + P20 + K0	7.8 × 10^−9^	4.7 × 10^2^	2.7 × 10^−8^	3.5 × 10^2^	1.4 × 10^−7^	2.8 × 10^2^
PS + P20 + K1	1.8 × 10^−9^	4.3 × 10^2^	1.4 × 10^−7^	5.5 × 10^2^	5.5 × 10^−9^	2.9 × 10^2^
PS + P20 + K2	2.0 × 10^−9^	4.1 × 10^2^	1.4 × 10^−7^	5.5 × 10^2^	5.3 × 10^−9^	2.7 × 10^2^
PS + P20 + K3	2.1 × 10^−9^	4.0 × 10^2^	1.3 × 10^−7^	5.4 × 10^2^	5.2 × 10^−9^	2.6 × 10^2^
PS + P20 + K4	2.3 × 10^−9^	3.8 × 10^2^	1.3 × 10^−7^	5.4 × 10^2^	5.0 × 10^−9^	2.3 × 10^2^
PS + P20 + K5	2.4 × 10^−9^	3.7 × 10^2^	1.3 × 10^−7^	5.4 × 10^2^	4.8 × 10^−9^	2.1 × 10^2^

P20 = 20 μL PBS, K1 = 1 μL *K12* (c_b_ = 0.23 × 10^9^ cells/mL), K2 = 2 μL *K12* (c_b_ = 0.45 × 10^9^ cells/mL), K3 = 3 μL*K12* (c_b_ = 0.65 × 10^9^ cells/mL), K4 = 4 μL *K12* (c_b_ = 0.88 × 10^9^ cells/mL), K5 = 5 μL *K12* (c_b_ = 1.26 × 10^9^ cells/mL).

## Appendix B

**Table A13:** The impedance amplitudes for the boron-doped BS biochip with low-concentration *K12* at 40 Hz, 400 Hz, and 4 kHz (Figure A1).

	|Z|(Ω) @ 40 Hz	|Z|(Ω) @ 400 Hz	|Z|(Ω) @ 4000 Hz
BS + W20 + K1	17956	16325	8732
BS + W20 + K2	17202	16088	8425
BS + W20 + K3	16871	15851	8321
BS + W20 + K4	16689	15122	8258
BS + W20 + K5	16198	14712	8104

W20 = 20 μL DI water, K1 = 1 μL *E. coli* (cb = 0.06 × 109/mL), K2 = 2 μL *E. coli* (cb = 0.11 × 109/mL), K3 = 3 μL *E. Coli* (cb = 0.16 × 109/mL), K4 = 4 μL *E. coli* (cb = 0.21 × 109/mL), K5 = 5 μL *E. coli* (cb = 0.25 × 109/mL).

**Table A14:** The impedance amplitude for the PS biochip with low-concentration *K12* at 40 Hz, 400 Hz, and 4 kHz. (Figure A1).

	|Z|(Ω) @ 40 Hz	|Z|(Ω) @ 400 Hz	|Z|(Ω) @ 4000 Hz
PS + W20 +K1	820	805	768
PS + W20 +K2	812	796	761
PS + W20 +K3	804	789	752
PS + W20 +K4	796	783	748
PS + W20 +K5	790	778	741

W20 = 20 μL DI water, K1 = 1 μL *E. coli* (c_b_ = 0.06 × 10^9^ cells/mL), K2 = 2 μL *E. coli* (c_b_ = 0.11 × 10^9^/mL), K3 = 3 μL*E. coli* (c_b_ = 0.16 × 10^9^/mL), K4 = 4 μL *E. coli* (c_b_ = 0.21 × 10^9^/mL), K5 = 5 μL *E. coli* (c_b_ = 0.25 × 10^9^/mL).
